# Replication Fork Reactivation in a *dnaC2* Mutant at Non-Permissive Temperature in *Escherichia coli*

**DOI:** 10.1371/journal.pone.0033613

**Published:** 2012-03-19

**Authors:** Boubekeur Saifi, Jean-Luc Ferat

**Affiliations:** 1 Centre de Genetique Moleculaire du CNRS, Gif Sur Yvette, France; 2 Universite de Versailles Saint Quentin, Versailles, France; University of Massachusetts Medical School, United States of America

## Abstract

Replicative helicases unwind double-stranded DNA in front of the polymerase and ensure the processivity of DNA synthesis. In *Escherichia coli*, the helicase loader DnaC as well as factors involved in the formation of the open complex during the initiation of replication and primosomal proteins during the reactivation of arrested replication forks are required to recruit and deposit the replicative helicase onto single-stranded DNA prior to the formation of the replisome. *dnaC2* is a thermosensitive allele of the gene specifying the helicase loader; at non-permissive temperature replication cannot initiate, but most ongoing rounds of replication continues through to completion (18% of *dnaC2* cells fail to complete replication at non-permissive temperature). An assumption, which may be drawn from this observation, is that only a few replication forks are arrested under normal growth conditions. This assumption, however, is at odds with the severe and deleterious phenotypes associated with a null mutant of *priA*, the gene encoding a helicase implicated in the reactivation of arrested replication forks. We developed an assay that involves an abrupt inactivation of rounds of synchronized replication in a large population of cells, in order to evaluate the ability of *dnaC2* cells to reactivate arrested replication forks at non-permissive temperature. We compared the rate at which arrested replication forks accumulated in *dnaC2 priA^+^* and *dnaC2 priA2* cells and observed that this rate was lower in *dnaC2 priA^+^* cells. We conclude that while replication cannot initiate in a *dnaC2* mutant at non-permissive temperature, a class of arrested replication forks (PriA-dependent and DnaC-independent) are reactivated within these cells.

## Introduction

The processivity of DNA replication requires a 5′→3′ replicative helicase - DnaB in *Escherichia coli* - to unwind double-stranded DNA in front of, and in interaction with, the replisome [1]. The hexameric DnaB protein forms a stable ring-shaped structure that needs to be opened prior to its placement on a single-stranded DNA. In *E. coli*, this function is ensured by DnaC, the helicase loader and a ring breaker [2], which remains stably bound to DnaB [3] until activation of the replicative helicase by the primase [4].

The replicative helicase needs to be loaded onto DNA at two different stages of DNA replication: the initiation of replication and the reactivation of arrested replication forks (RF). At the time of replication initiation, the complex DnaB_6_DnaC_6_ is recruited after the formation of the open complex [5] and involves the initiator protein, DnaA [6], and likely DiaA [7]. Other mechanisms are specified to reload the replicative helicase during RF reactivation. The most prominent pathway involves PriA and the primosomal proteins PriB and DnaT [8]. In this case, the 3′→5′ helicase activity specified by PriA is required to make available a sufficient length of single-stranded DNA to allow the assembly of the primosomal proteins and the subsequent loading of DnaB onto the lagging strand [9]. Other proteins, such as Rep, which also specifies a 3′→5′ helicase activity, and PriC may have important functions during the reactivation of arrested RF [8]–[10].

*dnaC2* is a thermosensitive mutant of the replicative helicase loader described as a slow stop mutant for DNA synthesis [11]: at non-permissive temperature, new rounds of replication cannot initiate while most ongoing rounds of replication continues through to completion [11]. The proportion of *dnaC2* cells in which replication is incomplete was estimated to be 18% at a non-permissive temperature of 38°C [12], which indicates that DnaC activity is not only indispensable for the initiation of replication but is also required during RF reactivation. Yet, the severe and deleterious phenotypes associated with a *priA2* null mutant, in which arrested RF cannot be reactivated (poor viability, UV sensitivity, phenotypically *rec^-^*,…) [13], appear far more severe than what would be expected if a mere 18% of RF were arrested during DNA replication. This discrepancy suggests that the cells, in which arrested RF were not reactivated, represent only a fraction of those in which RF were inactivated.

To establish whether some arrested RF are reactivated in *dnaC2* cells at non-permissive temperature, we designed an assay allowing us to compare the accumulation of arrested RF in *dnaC2* cells at non-permissive temperature in a *priA^+^* and in a *priA2* background. To facilitate the measure, we inactivated abruptly a large quantity of RF in a population of synchronized cells. We chose to inactivate RF with Novobiocin, a drug that inhibits type II topoisomerases and mainly Gyrase [14], after establishing that *priA2* cells were hypersensitive to Novobiocin. Gyrase eliminates the positive supercoils that accumulate in front of the RF and introduces negative supercoils ensuring the progression of the polymerase [15]. The accumulation of positive supercoils in front of the RF, when Gyrase is inhibited, halts the progression of the polymerase and eventually inactivates RF. We measured by flow cytometry the accumulation of inactivated RF in different genetic backgrounds and found that *dnaC2 priA2* cells accumulated 2.5 times more arrested RF than isogenic *priA^+^* cells at non-permissive temperature. This work led us to the identification of a new class of arrested RF - representing 60% of of them, whose reactivation depends on PriA but apparently not on DnaC activity. Implications in terms of DnaC2 activity at non-permissive temperature and in terms of the frequency of RF inactivation during normal growth are discussed.

## Materials and Methods

### Strains, chemicals and cultures

Strains used in this study and their genetic background are presented in Table 1. To increase the sensitivity of the cells to Novobiocin, a Δ*acrA* mutation was introduced in the strains used in this study [16]; AcrA is a component of an efflux pump that expels various drugs in the medium, reducing their intracellular concentration. In a Δ*acrA* mutant, the amount of Novobiocin required to inhibit type II Topoisomerase activity is lower than in *acrA^+^* cells [14], [17]. A *priA2* mutation is characterized by a high basal level of SOS induction [18]. Therefore, a *sfiA^−^* mutation was introduced in *priA2* strains to prevent filamentation [19]. *dnaC2* was P1 transduced from the original PC-2 strain [11]. A *mdoB*::Tn cassette [18] was first introduced within PC-2 to shuttle the *dnaC2* allele into CM735. The *dnaA46* strain is CM742 [20]. *priA2* cells were propagated with a pAM-*priA* plasmid until the time of experiment and eliminated according to the protocol described in [19].

**Table 1:** Strains and plamids used in this study.

*Strains*	*Genotype*	reference
BW25113	*lacIq rrnBT14 ΔlacZWJ16 hsdR514 ΔaraBADAH33*, *ΔrhaBADLD78*	[20]
CAG18430	*mdoB::Tn10*	[29]
CM735	*metE46*, *trp3*, *his4*, *thi1*, *galK2*, *lacY1* or *lacZ4*, *mtl1*, *ara9*, *tsx3*, *ton1*, *rps8* or *rps9*, *supE44 λ^−^*	[20]
JJC1398	AB1157 *sfiA11 priA2*::Kn/*pAM-priA*	[19]
JW0452	BW25113 *acrA*::Kn	[16]
JW0941	BW25113 *sfiA*::Kn	[16]
NK9069	CM735 *dnaA46*	[20]
PC2	*leu6 thyA47 dra3 str153 dnaC2 dnaT12*	[11]
REP1329	CM735 *dnaC2 mdoB*::Tn10	This study
REP1952	CM735 *acrA*::Kn	This study
REP2139	CM735 *ΔsfiA*(Kn^S^) *ΔacrA*(Kn^S^) *priA2*::Kn*/pAM-priA*	This study
REP1986	CM735 *dnaC2 mdoB*::Tn10 *ΔacrA*::Kn	This study
REP2364	REP2139 *dnaC2 mdoB*::Tn10	This study
REP2370	REP2139 *dnaA46 tna*::Tn10	This study
REP2031	REP1952 *dnaA46 tna*::Tn10	

*Plasmids*	*Description*	reference
*pAM-priA*	Plasmid, whose replication is under the control of P_lac_, carrying a WT copy of *priA*.	[19]

Cells were grown in minimal medium (K_2_HPO_4_ 10.5 g/l, KH_2_PO_4_ 4.5 g/l, (NH_4_)2SO_4_ 1 g/l, Sodium Citrate 0.5 g/l) complemented with MgSO_4_ 1 mM, CaCl_2_ 0.1 mM and with Glucose (0.2% w/v) as a carbon source. The procedure of cell cycle synchronization has been described elsewhere [21].

When required, the following chemicals were added (final concentration): L-Histidine (40 µg/ml), L-Tryptophane (40 µg/ml), L-Methionine (40 µg/ml), thiamine (1 µg/ml), Spectinomycin (60 µg/ml), Kanamycin (50 µg/ml), Tetracycline (15 µg/ml). Chemicals, antibiotics and amino acids were purchased from SIGMA and the culture reagents from DIFCO.

### Flow cytometry analysis

Cells analyzed by flow cytometry were previously fixed by adding 5 volumes of ethanol 70% per volume of sample. Before analysis, the cells were washed twice in filtered TE [Tris-HCl (pH 7.5) 10 mM, EDTA (pH 8) 1 mM] and resuspended in TE supplemented with RNase A and Propidium Iodide (10 µg/ml, each). A complete degradation of RNA was ensured by the incubation of the samples for 2 hours at 37°C prior to the cytometry analysis. Stained cells were excited at 532 nm with a Green NdYAG solid state laser. The light scattered by individual cells and the fluorescence emitted were detected, and amplified in a PARTEC Particle Analyzing System, PAS III. The forward scatter (FSC) gives a rough estimate of the cell mass, and the fluorescence signal emitted and recovered on the FL3 channel (>650 nm) was used to quantify the amount of DNA per cell. 100 000 cells were analyzed per run. Data were analyzed with the Flowmax software version 2.52.

### RF inactivation and RF reactivation

The fraction of cells containing one genome before (t0, α_0_), and 40 minutes after (t40 α_40_), the temperature downshift (initiation of replication), was measured on cytograms for each condition tested. The fraction of cells with active RF at t40 was calculated as ρ_40_ = 1−(α_40_/α_0_). In order to assess the kinetics of RF inactivation in response to Novobiocin, we calculated the ratio ρ_40_/ρ_40_^0^ for each time point and plotted the log value of these ratios over the time of incubation of Novobiocin at an inhibiting concentration. ρ_40_^0^ represents the proportion of cells with active RF at t40 that were not incubated with Novobiocin. The function Ln(ρ_40_/ρ_40_^0^) = f(t) is a straight line, whose slope (−κ) identifies the rate at which inactivated RF accumulate when the cells are incubated with Novobiocin. The fraction of RF reactivated in *dnaA46* and in *dnaC2* cells at non-permissive temperature was calculated as: 1−(κ_priA_^+^/κ_priA2_).

### Synchronization procedure

**Figure 1:**
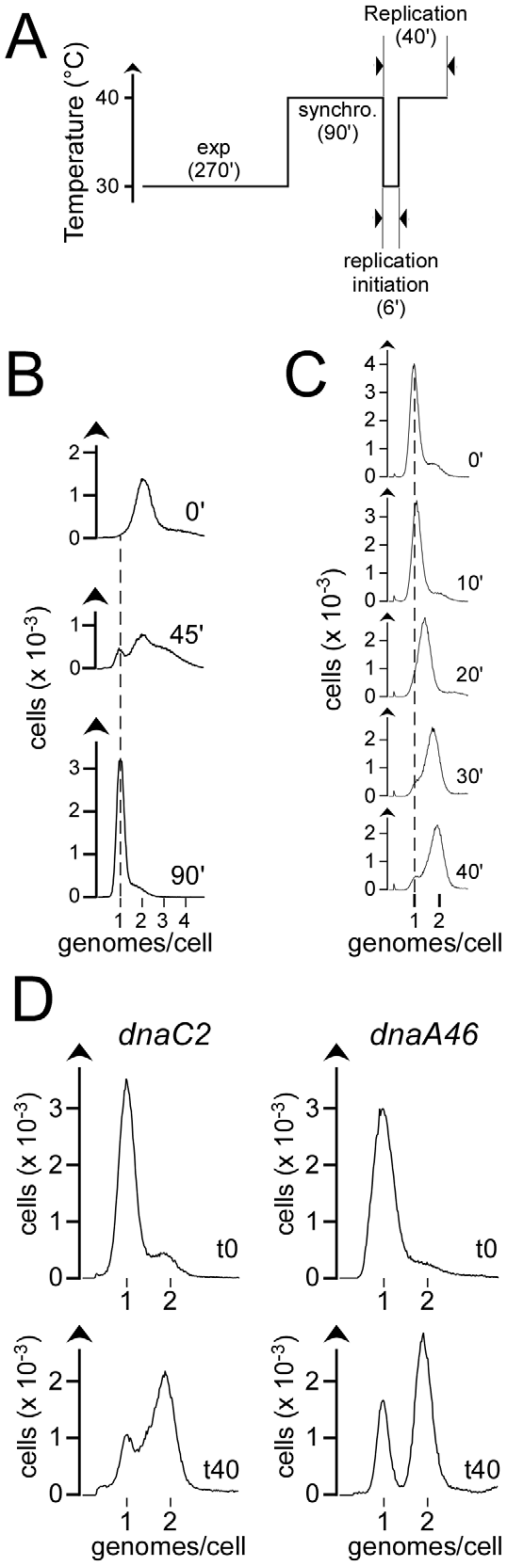
Experimental design. A – Synchronization procedure. A dilution of an overnight culture of *dnaC2* or *dnaA46* cells was grown during 3 generations at 30°C until exponential phase (exp) and then incubated at 40°C for 90 minutes (synchro) to synchronize the cells with respect to the initiation of replication. Replication was initiated by an abrupt downshift of the temperature of the culture to 30°C. 6 minutes after the temperature downshift, cultures were reincubated at 40°C to prevent the initiation of new rounds of replication. Samples were taken 40 minutes after initiation of replication.Time in minutes is indicated in brackets B – DNA histograms of a culture of exponentially growing *dnaC2* cells at 30°C before (0′) and after being shifted to 40°C for 45 (45′) and 90 minutes (90′). Similar DNA histograms were obtained with cultures of *dnaA46* cells (data not shown). After 90 minutes of incubation at non-permissive temperature, *dnaC2* and *dnaA46* cells are synchronized with respect to the initiation of replication. The dashed line indicates the position of the peak on a DNA histogram of stationnary phase cells (i.e., cells containing one genome). C – DNA histograms of *dnaC2* cells harvested at different time points after the temperature downshift (0′, 10′, 20′, 30′ and 40′). The dashed line indicates the position of the peak on a DNA histogram of stationnary phase cells (i.e., cells containing one genome). D – DNA histograms of synchronized *dnaC2* (left) and *dnaA46* cells (right) before initiation of replication (t0) and 40 minutes after replication initiation (t40). Replication was initiated by shifting abruptly the temperature from 40° to 30°C. After 6 minutes of incubation at a permissive temperature for the initiation of replication (30°C), the cells were brought back to 40°C. 40 minutes after initiation of replication, cells with active RFcontain around two genomes.

Overnight cultures of *dnaC2* or *dnaA46* cells were diluted in fresh MMA Glucose medium and grown at permissive temperature (30°C) for 3 generations (doubling time of 90 min.) until reaching log phase (OD_550_ of 0.1). The cultures were then transferred into a shaking water bath preset at 40°C for 90 minutes to synchronize the cells with respect to replication initiation [21]. Replication was initiated by shifting abruptly the cultures to 30°C for 6 minutes. This operation was performed by the addition of an equal volume of 20°C-prewarmed fresh medium to the culture, followed by the incubation of the culture at 30°C in a shaking water bath. 6 minutes after the temperature downshift, the cells were reincubated at 40°C to prevent the initiation of additional rounds of replication (Figure 1A).

## Results

### Assessing the fraction of cells with active RF

The appreciation of the ability of *dnaC2* cells to reactivate arrested RF requires a precise evaluation of the proportion of cells with active RF in a given population, which is made possible by flow cytometry. The incubation of exponentially growing *dnaA46* cells – carrying a thermosensitive allele of the gene encoding the initiator protein - or *dnaC2* cells at a non-permissive temperature blocks the initiation of replication but neither ongoing rounds of replication, which continue through to completion, nor cell division. During an incubation of 90 minutes at a non-permissive temperature of 40°C, a population of *dnaC2* or of *dnaA46* cells is progressively enriched in cells containing a single genome (Figure 1A and 1B). After 90 minutes of such a treatment, most cells contain one genome (cells with two or more genomes amount to a mere 10 to 15%); the cells are synchronized with respect to replication initiation (Figure 1B). At this stage, a short (6 minutes) and abrupt downshift of temperature to 30°C - of cultures of *dnaC2* and *dnaA46* cells pre-incubated for 90 minutes at 40°C - results in a synchronous initiation of replication in a large proportion of cells. The quantity of DNA (followed over FL3) in cells that initiated replication shifts over time from 1 to 2 genomes (Figure 1C). 40 minutes after the temperature downshift (t40), which corresponds to the period of time required to complete a round of replication (data not shown), *dnaC2* and *dnaA46* cells that initiate replication contain around 2 genomes. These cells are easily identifiable as a peak on DNA histograms (Figure 1D).

It is noteworthy that the peak of replicating cells is broader in a *dnaC2* background than in a *dnaA46* background (Figure 1D). The peak of *dnaC2* replicating cells overlaps partially with that of cells containing one genome while the peaks of *dnaA46* cells with one and with two genomes are relatively narrow and well separated (Figure 1D). From cell to cell, the quantity of DNA replicated in *dnaC2* cells during 40 minutes is more variable than in *dnaA46* cells. Assuming that the elongation rate of the DNA polymerase is not altered in a *dnaA46* and in a *dnaC2* background, the broader shape of the peaks observed with *dnaC2* cells at t40 likely reflects the fact that within this genetic background, rounds of replication were arrested and not reactivated at non-permissive temperature. Our data are therefore consistent with previously reported results (Maisnier-Patin et al, 2001), but still do not refute the possibility that some arrested RF be reactivated in *dnaC2* cells at non permissive temperature.

To shed light on this matter, we developed a method allowing us to measure the fraction of cells with active RF within a population.

**Figure 2:**
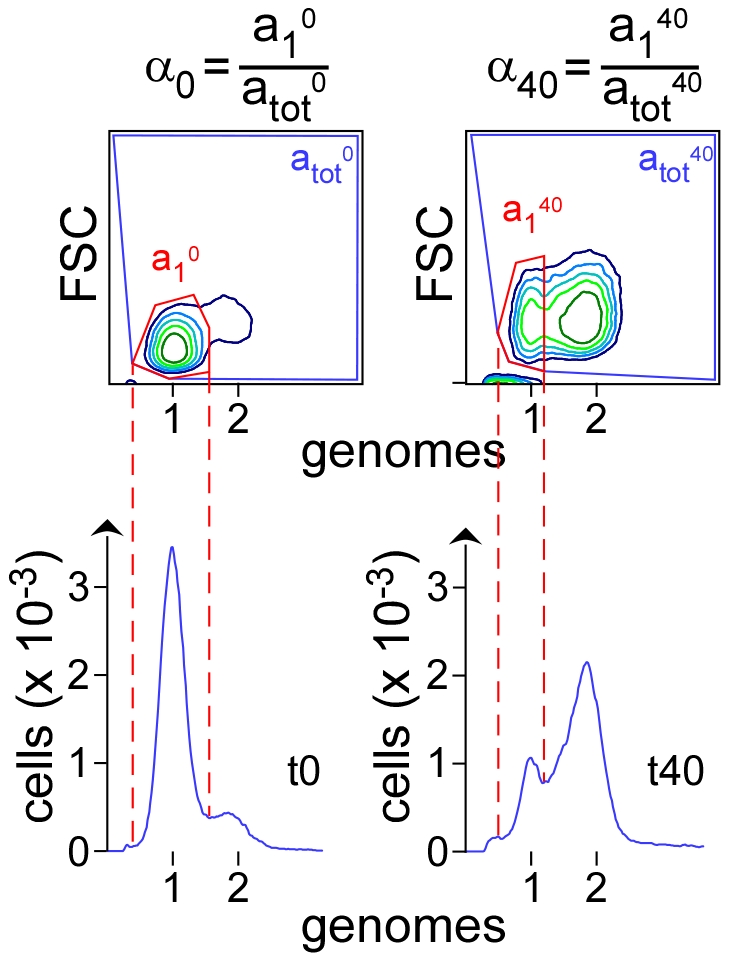
Assessment of the proportion of cells with active RF in a population of synchronized cells. Contour plot of cytograms (FL3 [DNA] vs. FSC [mass], top) and DNA histograms (bottom) of *dnaC2* synchronized cells before (t0) and 40 minutes after replication initiation (t40). Cells with one genome at t0 and at t40 (a_1_^0^ and a_1_^40^) are circled in red on cytograms. The corresponding peaks are delineated by dashed lines on DNA histogram. The total amount of cells within the samples (a_tot_^0^ and a_tot_^40^) is circled in blue on cytograms. The formula above the cytograms were used to calculate the proportion of cells with one genome at t0 and at t40 (α_0_ and α_40_) and then the proportion of cells with active RF (ρ_40_) (Materials and Methods).

Synchronized cells that initiated and completed a round of replication accumulate within the peak at 2 genomes per cell (Figure 2), while those in which replication did not initiate accumulate within the peak at one genome per cell. Cells in which 1 or 2 RF were arrested - before completion of replication - and not reactivated accumulate in the valley between 1 and 2 genomes per cell.

The drift of the peak on DNA histograms of cells harvested before, and 10 minutes after, the temperature downshift is almost negligible (Figure 1B). Therefore, cells in which both RF were arrested within 10 minutes after the initiation of replication and not reactivated, accumulate with those that did not initiate replication. Hence, and in order to assess the ability of *dnaC2* cells to reactivate arrested RFa large proportion of RF was transiently inactivated soon after initiation of replication in a synchronized population of cells. Then, the cells were brought back to growth conditions permissive with respect to replication. Under such experimental conditions, cells in which replication did not initiate and those in which both RF were arrested - during the inactivation procedure - and not reactivated, accumulate within the peak at one genome d (Materials and Methods). , We turned to the analysis of cytograms, in which the DNA content (given by the FL3) is plotted over the FSC, which gives a rough estimate of cell mass,to delineate more precisely the fraction of cells with one genome (Figure 2). Given this starting situation, the proportion of cells undergoing replication under a given condition (ρ_40_) can be extracted from the fraction of cells with one genome at t0 (α_0_) and t40 (α_40_) (Figure 2, Materials and Methods).

### Inactivating RF with Novobiocin

**Figure 3:**
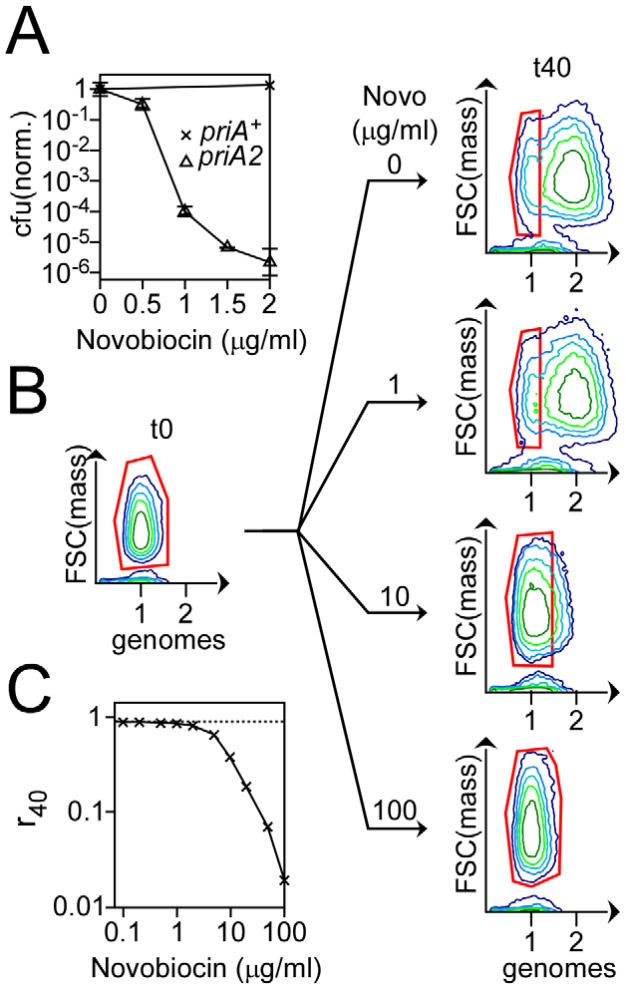
Effect of Novobiocin on RF inactivation. A – *priA^+^* (x) and *priA2* cells (Δ) were grown to log phase in minimal Glucose medium, diluted and plated on minimal Glucose plates to which Novobiocin was added. Colonies were counted after 3 days of incubation at 30°C, normalized to the cfu calculated in absence of Novobiocin and plotted over the concentration of Novobiocin. Error bars indicate the standard deviation around the mean in three independent experiments. B – Contour plots of *dnaC2* cells before replication initiation (t0) and 40 minutes after initiation of replication (t40). Cells that initiated replication were incubated 10 minutes after initiation of replication with different concentrations of Novobiocin for 30 minutes. The final concentration of Novobiocin (in µg/ml) is indicated above the arrows. Cells containing one genome at t0 or at t40 are circled in red. C – The proportion of *dnaC2* cells with active RF under each condition tested (ρ_40_, Materials and Methods) was plotted over the range of concentrations of Novobiocin tested.

We developed a procedure to generate a large quantity of arrested RF in cultures of synchronized cells to assess the capacity of *dnaC2* cells to reactivate RF at non-permissive temperature. We decided to target Gyrase because PriA was shown to be essential in gyrase point mutants, revealing a high rate of RF inactivation in these strains; the mediocre activity specified by Gyrase mutant proteins and the resulting accumulation of positive supercoils in front of the polymerase was proposed to be responsible for the high rate of RF inactivation [19]. Novobiocin is a Gyrase inhibitor. Topo IV, the topoisomerase implicated in the resolution of (pre)catenated DNA that accumulates behind the RF [22], is also targeted by Novobiocin. Yet, Topo IV is much less sensitive to Novobiocin than Gyrase [14], implying that Gyrase is the primary target of Novobiocin. We verified whether the Novobiocin-induced inhibition of Gyrase could inactivate RF by assessing the sensitivity of a *priA2* mutant – a mutant in which most arrested RF is not reactivated – to Novobiocin. *priA^+^* and *priA2* mutant cells were plated on minimal medium in which Novobiocin was added at different concentrations. At a concentration of Novobiocin of 1 µg/ml, the colony-forming unit (cfu) of *priA2* cells was around 1/10,000 that measured without Novobiocin. In contrast, the viability of *priA^+^* cells was unaffected by Novobiocin up to a concentration of 2 µg/ml (Figure 3A). This result indicates that Novobiocin induces the inactivation of RF.

*dnaC2* cells synchronized with respect to replication were treated 10 minutes after replication initiation and during 30 minutes with Novobiocin at different concentrations and then analyzed by flow cytometry (Figure 3B, Materials and Methods). Under these conditions, the peak centered at one genome identifies the cells that did not initiate replication and those in which the two RF were inactivated in response to Novobiocin. The proportion of cells with active RF was measured (Materials and Methods) for each condition (Figure 3B) and then plotted over the range of concentrations of Novobiocin tested (Figure 3C). Up to a concentration of 2 µg/ml, Novobiocin has virtually no effect on the proportion of cells with active RF (Figure 3C). In contrast, the proportion of cells with active RF was significantly reduced when the concentration of Novobiocin was at least 5 µg/ml (Figure 3C). We chose to generate arrested RF by treating cultures of cells with Novobiocin at a concentration of 10 µg/ml (an “inhibiting” concentration) because a large quantity of RF are inactivated at this concentration of drug; the fraction of cells with active RF was estimated to be less than 40% (Figure 3C). A concentration of 10 µg/ml of Novobiocin was also chosen because a 10 fold dilution brings the drug to a concentration at which the proportion of cells with active RF is merely 1.9% less than that measured in cultures of untreated cells (Figure 3C).

### RF are reactivated in dnaC2 cells at non-permissive temperature

**Figure 4:**
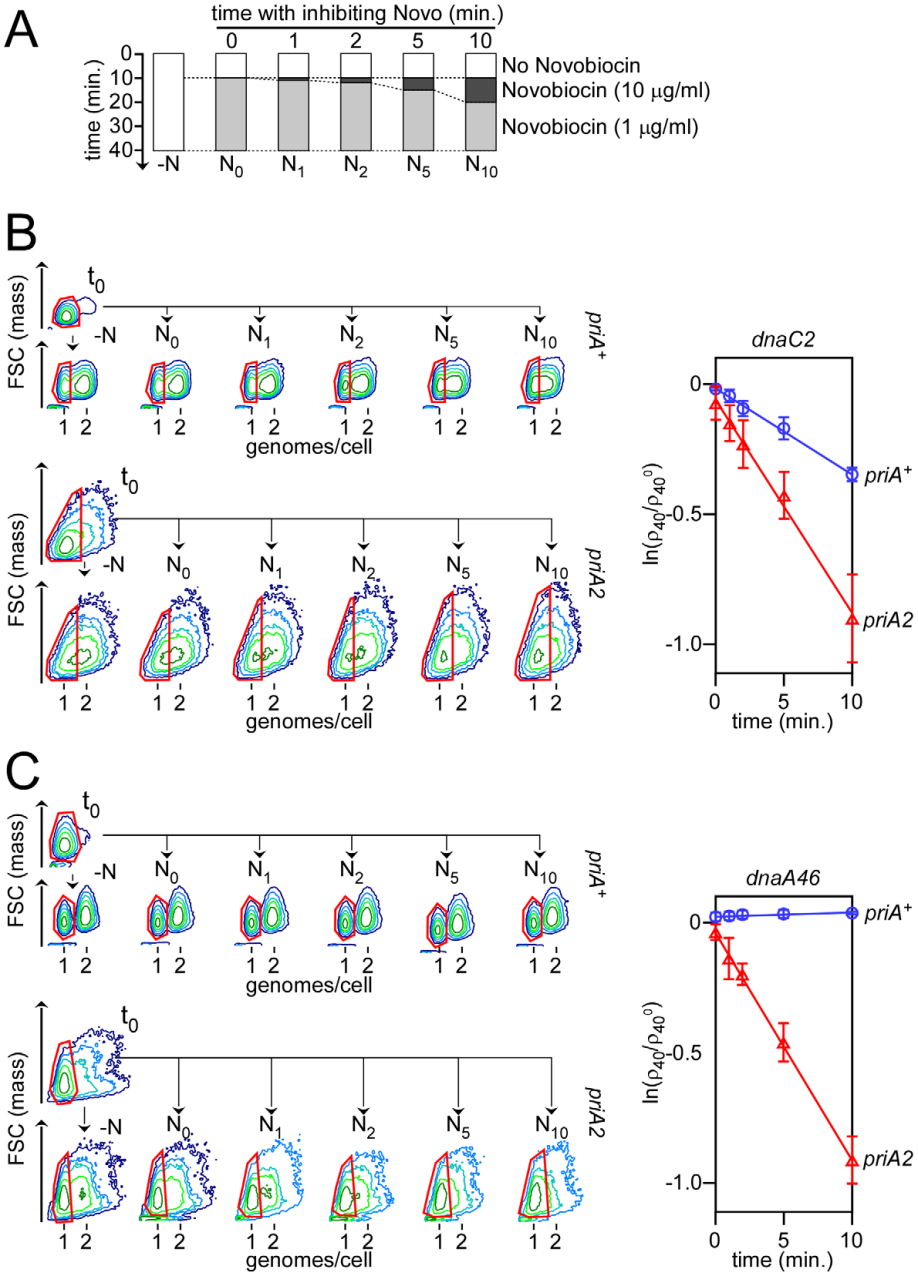
RF reactivation in *dnaC2* cells at non-permissive temperature. A – Schematic description of the experimental procedure. Replication is initiated synchronously at t0. 10 minutes after replication initiation, Novobiocin is added to the cultures at an inhibiting concentration (dark grey) before being brought back to a permissive concentration (light grey) until the end of the experiment. A control sample, not treated with Novobiocin, (−N) was also analyzed. Samples incubated with Novobiocin (N_i_) are identified by the time ‘i’ (in minutes) of incubation at a concentration of Novobiocin of 10 µg/ml. B – Contour plots of *dnaA^+^ dnaC2* cells before (t0) and after initiation of replication (−N, N_0_, N_1_, N_2_, N_5_ and N_10_) in *priA^+^* (top) and *priA2* cells (bottom). The fraction of cells with active RF (ρ_40_) under each condition tested was normalized to that of cells that were not incubated with Novobiocin (ρ_40_^0^), and the logarithm value of these ratios was plotted over the time of incubation with Novobiocin at 10 µg/ml (plot on the right). Circles (*priA^+^*) and triangles (*priA2*) identify the average value for a given time point, and error bars correspond to the standard deviation around the mean in three independent experiments. C –Same as B except that the cells analyzed are *dnaA46 dnaC^+^* and that error bars for *priA^+^* cells correspond to the standard deviation around the mean in two independent experiments.

Novobiocin was added to synchronized cultures of *priA^+^* and *priA2* cells to a final concentration of 10 µg/ml, 10 minutes after replication initiation and for a length of time ranging from 1 to 10 minutes. Then the cultures were diluted to bring Novobiocin to a concentration at which the drug has an insignificant effect on Gyrase activity in *dnaC2* cells (1 µg/ml). 40 minutes after initiation of replication, samples of cells were taken, fixed and processed for flow cytometry analysis (procedure summarized in Figure 4A). The experiment was performed in a *dnaC2* (Figure 4B) and in a *dnaC^+^* background (Figure 4C). In the latter case, cells were synchronized with *dnaA46*.

It was shown in *dnaC2* cells that the inactivation of one RF does not affect the fate of the other RF present in the cell [12]. Hence, we measured the rate at which cells with two inactive RF were generated, which corresponds to the rate at which the last RF was inactivated, to estimate the rate of RF inactivation in response to Novobiocin. We followed the fraction of cells with active RF for each strain tested over the time of incubation with Novobiocin at an inhibiting concentration (Materials and Methods). ρ_40_ was measured for each time point, divided by ρ_40_^0^ (where ρ_40_^0^ is the fraction of cells with active RF in the absence of Novobiocin) and the logarithm value of these ratios were plotted over the time of incubation of the cultures at an inhibiting concentration of Novobiocin (Figure 4B and 4C). For each strain, the distribution of these values fits best with a linear distribution (coefficient of determination, R^2^, above 0.99), which indicates that RF inactivation in response to Novobiocin is a first-order reaction. For each series of data, the linear regression was assessed and the slope (−κ) was extracted (Figure 4B and 4C). κ represents the rate at which inactivated RF accumulate during the experiment. The values of κ calculated in a *dnaC2 priA2* (0.084±0.012 min^−1^) and in a *dnaC^+^ priA2* background (0.09±0.009 min^−1^) are not significantly different, which indicates that *priA2* is epistatic to *dnaC2*. Strikingly, the value of κ calculated in a *dnaC2 priA^+^* background (0.033±0.004 min^−1^) is much lower than that calculated in a *dnaC2 priA2* background. This result indicates that *dnaC2 priA^+^* cells accumulate less arrested RF than *dnaC2 priA2* cells in response to Novobiocin.

## Discussion

The primary result of this study is that *dnaC2 priA2* cells accumulate more inactivated RF at non-permissive temperature than *dnaC2 priA^+^* cells when the cells are treated with Novobiocin, a drug that inhibits Gyrase. Two points, however, need further clarification in order to conclude definitely from these data that *dnaC2* cells can reactivate arrested RF at non-permissive temperature. The first point regards the direct link that we drew between the inhibition of Gyrase and the inactivation of RF. We clarified this point by plating *priA2* and *priA^+^* cells on a medium supplemented with Novobiocin. The rationale behind this experiment was based on the requirement of the helicase activity specified by PriA and the following recruitment of the primosomal proteins for the reactivation of arrested RF [9]. Another pathway – driven by PriC and independent of PriA – was deduced from the synthetic lethality associated with a double mutant *priA priC* [8]. Yet, the absence of phenotype attributable to a *priC* single mutant led to the assumption that the PriA-driven mechanism was the major RF reactivation pathway. Thus and despite the existence of an alternative pathway for RF reactivation, we reasoned that the inhibition of Gyrase by Novobiocin, if it results in the inactivation of RF, ought to reduce dramatically the viability of *priA2* mutant cells at low concentration of drug. We established indeed that *priA2* cells are much more sensitive to Novobiocin than *priA^+^* cells (Figure 3A) and concluded that the Novobiocin-induced inhibition of Gyrase leads to RF inactivation. The second point concerns the rate at which RF are inactivated in *dnaC2* and in *priA2* cells in response to Novobiocin. This point requires a clarification as well because a faster rate of RF inactivation in *priA2* than in *priA^+^* cells, or the destabilization of active RF caused by the absence of the PriA protein could alternatively explain the larger proportion of cells with arrested RF in a *dnaC2 priA2* than in a *dnaC2 priA^+^* background. This hypothesis, however, may be excluded since PriA interacts with DNA after RF inactivation - and not before [9]. We may also exclude the possibility that a Δ*sfiA* mutation – which we introduced in *priA2* cells to prevent the *priA2*-induced SOS response to inhibit cell division – modulate the stability of RF or the rate at which they are inactivated since we established that the rate, at which inactivated RF accumulate, was identical in *dnaC2 sfiA^+^* and in *dnaC2 sfiA^−^* cells (data not shown). An additional point may be made with regards to the SOS response: is it possible that the SOS response, which is induced in *dnaC2* cells at non-permissive temperature [23], modifies the fate of ongoing rounds of replication? In this respect, an over-stabilization of active RF in *dnaC2* cells in response to the induction of the SOS response may be excluded since this response is induced in *priA2* cells as well. We therefore conclude that the same quantity of RF was inactivated in *dnaC2 priA* ^+^ and in *dnaC2 priA2* cells during the Novobiocin treatment.

We considered also the possibility that some inducible Stable DNA replication (iSDR) – induced during the SOS response [24] – be initiated in *dnaC2* cells and misinterpreted as RF reactivation. For this hypothesis to be valid, however, one would have to assume that the replicative helicase can be loaded in *dnaC2* cells at non-permissive temperature as well, since DnaC and PriA activities were shown to be required for the initiation of iSDR [25]–[26].

The reduced cfu of *priA2* cells grown with Novobiocin at a concentration of 1 µg/ml indicates that RF are also inactivated at this concentration of drug (Figure 3A). Hence, one may argue that in addition to the RF that were inactivated during the incubation of the cells with Novobiocin at a concentration of 10 mg/ml, other RF were inactivated during the so-called “permissive conditions” of our experiment, i.e., after Novobiocin was diluted to a concentration of 1 µg/ml. The quantity of inactivated RF was estimated. The proportion of cells with active RF was merely reduced by 1.9 and 4.5% in *dnaC2 priA^+^* and *dnaC2 priA2* cells, respectively, after a 30 minutes incubation of the cells with Novobiocin at a concentration of 1 µg/ml (condition N_0_ in Figure 4). In contrast, the proportion of *dnaC2 priA^+^* and *dnaC2 priA2* cells with active RF was reduced by 30 and 60%, respectively, after a 10 minutes incubation of the cells with Novobiocin at a concentration of 10 µg/ml. Thus, and although the objection is legitimate, we considered the fraction of cells in which RF were inactivated during the incubation of the cells with Novobiocin at 1 µg/ml to be low enough to be insignificant. Hence, the calculation of the proportion of cells with active RF after 40 minutes of treatment with Novobiocin, as presented here, is appropriate and meaningful to determine whether arrested RF were or were not reactivated in *dnaC2* cells at non-permissive temperature.

*dnaC2 priA2* and *dnaC^+^ priA2* cells accumulate inactivated RF at a similar rate in response to Novobiocin (0.084 and 0.09 min^−1^ in *dnaC2 priA2* and *dnaC^+^ priA2*, respectively), which indicates that the rate at which *priA2* cells accumulate inactivated RF is independent of the allele of *dnaC*. Under the same experimental conditions, *priA^+^ dnaC2* cells accumulate inactivated RF at a much lower rate (0.033 min^−1^), while inactivated RF were undetectable in *priA^+^ dnaA46* cells;the reactivation of a fraction of arrested RF is dependent on DnaC, in agreement with previous results [12]. Altogether, these results indicate that among the arrested RF, 40% need active DnaC for their reactivation, while 60% do not. (Materials and Methods). Granted that around 18% of *dnaC2* cells fail to complete replication when cultivated at non-permissive temperature (i.e., cells in which arrested RF were not reactivated) [12], and assuming that the efficiency of RF reactivation in *dnaC2* cells at non-permissive temperature is the same with and without Novobiocin, we may concludethat around 45% of replications are arrested before completion, under normal growth conditions.

This study raises new questions about the activity and the function specified by DnaC2 at non-permissive temperature. Can DnaB be loaded onto DNA without the assistance of DnaC? The replicative helicase is extremely stable as a hexameric ring and DnaC is referred to as a ring breaker in *E. coli* because it catalyzes the opening of the ring, which is required to place the helicase onto single-stranded DNA [2]. Thus, the loading of DnaB on DNA without the help of a loader may be excluded. Is it possible, however, that another factor specify the function of loading the replicative helicase onto DNA? During the replication of the bacteriophage lambda, for example, the loading of DnaB at the origin of replication of the phage is ensured by the phage protein λP and not by DnaC [27]. Our strain is devoid of lambda [20] and the inspection of its genome (through BLAST) did not reveal the presence of close or distantly related copies of the lambda P gene. Yet, we cannot exclude that an alternative replicative helicase loading system be specified in *E. coli*. Since *dnaC* is an essential gene, however, such an alternative replicative helicase loading system ought to work specifically during the reactivation of arrested RF and not during the initiation of replication.

Is DnaC systematically required to reactivate arrested RF? If DnaB were still present on arrested RF, for example, its reactivation should not require DnaC. Such a situation should not require PriA either, because the very function of PriA is to assist the loading of the replicative helicase onto DNA [9]. Thus, this hypothesis may be excluded. We may instead consider that DnaC2 is active for reloading DnaB at arrested RF and at non-permissive temperature. Yet, if DnaB can be loaded at arrested RF by DnaC2 at non-permissive temperature, why cannot DnaB be loaded at *oriC* by DnaC2 during the initiation of replication? The fact that different partners are involved in the recognition of the helicase complex is most certainly part of the answer. During replication initiation at *oriC*, the replicative helicase complex interacts directly with DnaA [6], while the replicative helicase is presented to a complex composed of primosomal proteins during the reactivation of the RF [9]. It is tempting to speculate that the aptitude of the DnaC2 mutant protein to load the replicative helicase at non permissive temperature reflects indirectly a property acquired by the helicase loader through evolution. While RF reactivation is vital for the cell and has to be ensured by any means, the blockage of the loading of the replicative helicase during replication initiation is not deleterious and may – in addition to the already known activities regulating this stage [28] - bring an additional level of control for the cell to verify that the conditions are appropriate to initiate replication of the chromosome. Thus, it is possible that structural peculiarities of the primosomal complex, specific interactions between the primosomal complex and the helicase complex, or even an as yet unknown additional factor, facilitate specifically the loading of the helicase during RF reactivation.
